# [Retracted] GDC-0152 attenuates the malignant progression of osteosarcoma promoted by ANGPTL2 via PI3K/AKT but not p38MAPK signaling pathway

**DOI:** 10.3892/ijo.2025.5827

**Published:** 2025-12-01

**Authors:** Lin Yang, Taipengfei Shu, Yingjian Liang, Wenguang Gu, Chunlei Wang, Xuanhe Song, Changdong Fan, Wenbo Wang

Int J Oncol 46: 1651-1658, 2015; DOI: 10.3892/ijo.2015.2872

Following the publication of the above paper, it was drawn to the Editor's attention by a concerned reader that, for the MTT assay experiments shown in Fig. 2A on p. 1655, the GDC-0152/ANGPTL2 panel appeared to overlap with the ANGPTL2 panel, albeit the panel had been rotated through 180°; moreover, the magnification of the right-hand panel was very different, creating an impression that the ANGPTL2 panel showed more cells. Upon analyzing the data independently in the Editorial Office, it came to light that that certain of the flow cytometric data in Fig. 2B and the nuclear staining experiments in Fig. 2C were strikingly similar to data in other articles written by different authors at different research institutes that had already been accepted for publication elsewhere. Owing to the fact that the contentious data in the above article had already been published prior to its submission to *International Journal of Oncology*, the Editor has decided that this paper should be retracted from the Journal. The authors were asked for an explanation to account for these concerns, but the Editorial Office did not receive a reply. The Editor apologizes to the readership for any inconvenience caused.

